# Exploring factors influencing patient choice in outpatient ophthalmology provider in the North London region: a patient survey

**DOI:** 10.1136/bmjhci-2024-101360

**Published:** 2025-10-29

**Authors:** Rishi Ramessur, Roxanne Crosby-Nwaobi, Claire Lovegrove, Julia Theodossiades, Rachel Thomas, Estelle Ioannidou, Radhika Rampat, Peter B M Thomas

**Affiliations:** 1 Moorfields Eye Hospital NHS Foundation Trust, London, UK; 2 Institute of Global Health Innovation, Imperial College London, London, UK; 3 Royal Free London NHS Foundation Trust, London, UK; 4 NIHR Biomedical Research Centre for Ophthalmology, Joint Library of Ophthalmology Moorfields Eye Hospital and UCL Institute of Ophthalmology, London, UK

**Keywords:** Patient Involvement, Patient-Centered Care, Hospital-Patient Relations, Consumer health informatics, Access to Information

## Abstract

**Objectives:**

To explore factors important to patients when choosing a secondary care provider.

**Methods:**

A survey was distributed to 376 participants at Moorfields Eye Hospital comprising both free-text responses and Likert scale elements. Word frequency analysis was applied to free-text responses, and K-means analysis to identify clusters from Likert responses.

**Results:**

Reputation, expertise, quality of care and clinical outcomes were the most important factors driving patient preference—more so than practicalities such as travel, ease of access, parking and proximity to healthcare provider. One of two identified clusters of patients appeared to place higher value on societal benefits (eg, sustainability, carbon footprint minimisation, cost-efficiency for National Health Service (NHS)) than the other.

**Discussion:**

The current NHS approach of highlighting travel distance, wait times and Care Quality Commission (CQC) ratings when presenting choice is inadequate to support informed choice of provider. Further work in a more representative cohort and exploration of real-world patient decisions is warranted.

## Introduction

 Patient autonomy is a fundamental principle of care, yet its role in healthcare provider choice remains underexplored.^1^ Existing studies identify important factors relating to provider structure (accessibility, distance, staff qualifications, waiting times and service quality), processes (clinicians’ communication, information provision) and providers’ outcomes.^2^ However, since a 2012 scoping review, few studies have explored this, leaving gaps in understanding how modern healthcare challenges shape decision-making.^2^

While many studies underscore methodical and systematic processes in patient decision-making ^2^, their complexity is often simplified, particularly in retrospective analyses that risk assumptions about motivations. Additionally, influencing factors can vary depending on the decision-making context, yet few studies examine real-life choices.^2^

This study explores patient choice in a National Health Service (NHS) ophthalmology setting, assessing factors influencing provider preferences among patients and healthy volunteers affiliated with Moorfields Eye Hospital (MEH), London. This exploratory analysis will inform future work into patient preferences in a larger cohort referred via the North Central and North East London Integrated Care System Single Point of Access referral pathway.

## Methods

Patients were identified and contacted using the Moorfields Research Network’s ROAM (Research Opportunities at Moorfields) tool and invited to answer eighteen 5-scale Likert questions using SmartSurvey. Nine open-ended questions let respondents highlight factors beyond the Likert options (online supplemental appendix 1). Questionnaire design, refined through multidisciplinary consensus and peer feedback, incorporated NHS Choices system criteria and literature review.^3^

Free-text responses were lemmatised and stopwords removed. Word-mapping converted common abbreviations (eg, ‘info’) to full form (eg, ‘information’). Additional common words irrelevant to thematic analysis were removed, along with references to any particular healthcare provider. Word frequency was then calculated for each free-text question and overall.

Likert-scale responses were analysed using descriptive statistics and visualised with stacked bar graphs. K-means clustering was applied to Likert responses to see if respondents grouped into distinct clusters. Principal component analysis reduced 5-point Likert data to a low-dimensional space, and exploratory analysis determined the optimal number of clusters. Feature importance was assessed by examining each question’s contribution to cluster centroids. Mean and absolute differences in feature contribution across clusters were analysed to determine distinctive features (questions).

Python-3 packages on Jupyter Notebook were used for data analysis and visualisation.

## Results

376 respondents completed questionnaires from 13 January to 15 January 2024. The five most common words across free-text questions were ‘care’ (n=316), ‘reputation’ (n=201), ‘time’ (n=183), ‘expertise’ (n=165) and ‘experience’ (n=136). ‘Transport’ ranked 8th (n=131), ‘quality’ 9th (n=123), ‘information’ 10th (n=123), ‘travel’ 11th (n=122), ‘waiting’ 13th (n=110), ‘access’ 14th (n=102) and ‘location’ 22nd (n=76). Online supplemental figure 1 displays the 10 most common words across free-text questions. Online supplemental figure 2 presents a word cloud for question 1 responses.

**Figure 1:**
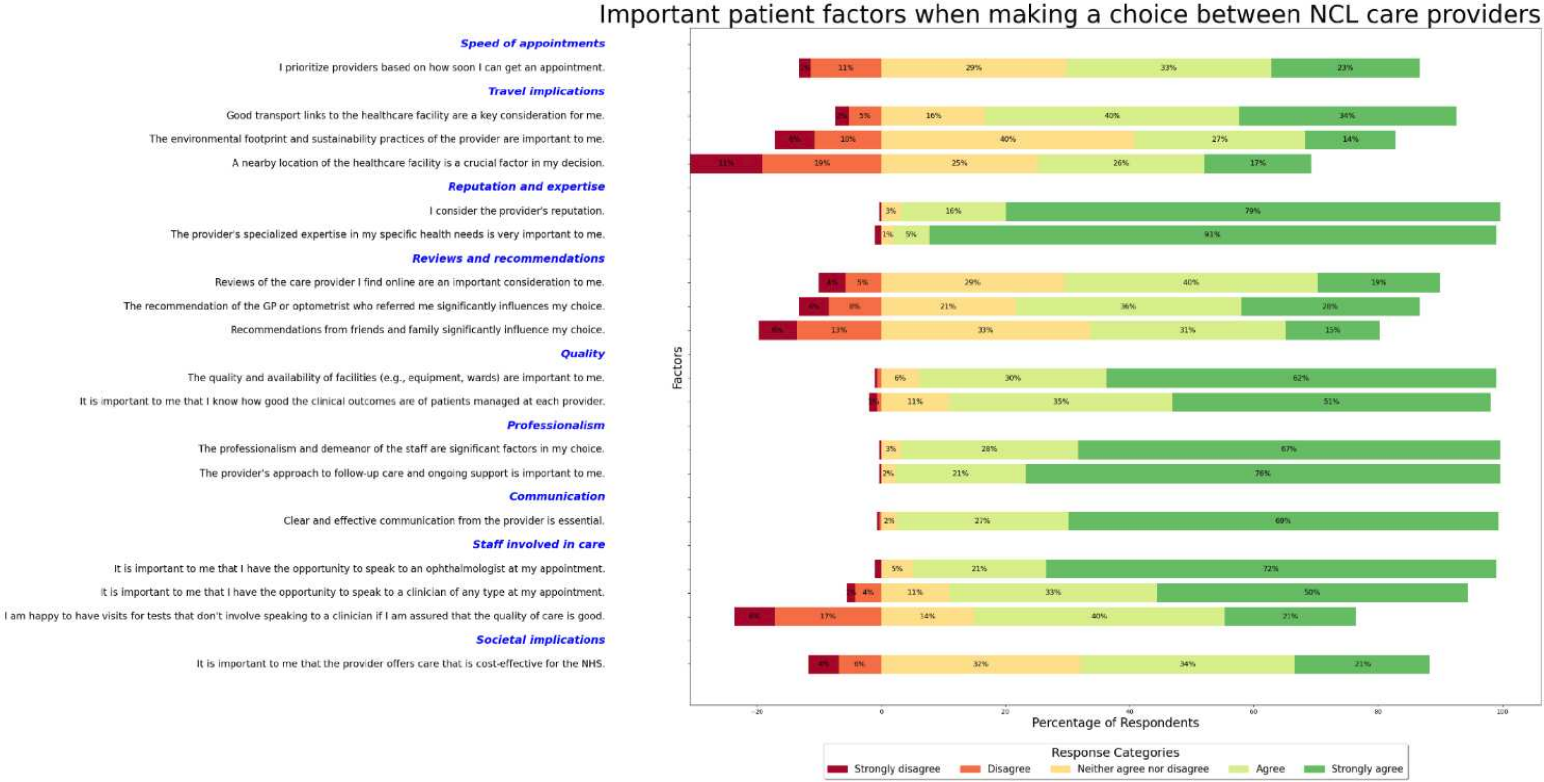
Responses by percentage to questions exploring factors that influence patient choice between ophthalmology providers. ‘Strongly disagree’ and ‘Disagree’ were assigned negative percentages. Questions were grouped by category for visualisation ease. Bars within categories are ordered ascending by percentage of respondents who disagreed or strongly disagreed with question statements. GP, general practitioner; NHS, National Health Service; NCL, North Central London.

Figure 1 displays a stacked bar chart of percentage responses to each Likert question option. Reputation, expertise, professionalism, communication, quality of facilities, clinical outcomes and seeing an ophthalmologist appear to be most important in influencing patients’ decisions.

Comparatively fewer patients feel that proximity, friend/family/general practitioner (GP) recommendations, environmental footprint and waiting times are important. Patients prefer speaking to clinicians at appointments, though many are happy with a visit comprising diagnostic testing only.

Exploratory analysis identified that two clusters most aptly describe the data. Important questions across both pertain to provider reputation, specialised expertise, facilities quality and availability, professionalism, communication, seeing an ophthalmologist and knowing clinical outcomes. Questions that differentiate clusters pertain to provider location, environmental footprint and cost-effectiveness for the NHS.

## Discussion

In our study, reputation, expertise, quality of care and clinical outcomes are seemingly most important to patients in choosing an ophthalmology provider—more so than travel, ease of access, parking and proximity to healthcare provider. These findings align with broader healthcare themes^2^ and a nearby survey of GPs and patients showing that patients prioritise reputation and expertise over waiting times and location, contrary to GPs’ preconceptions.^4^

The study’s multiple methodologies all lead to similar conclusions, enhancing the reliability of conclusions drawn. The selectively sampled Moorfields Research Network population allowed for quick exploratory analysis that will inform future work. However, participants have chosen to engage with a Moorfields Research Network and may therefore favour MEH. Nonetheless, MEH represents over 10% of UK ophthalmology patients and participant recruitment spans its network of 21 sites—including both district hospitals and the specialist hospital, so we cannot discount their feedback.

Findings from London’s geographically densely concentrated healthcare landscape may not apply to less urban areas. Geographical location, social status and patient choice are intricately linked, with perceptions about healthcare quality and importance of choice itself seemingly shaped by an area’s status.^5^ A deeper understanding of patient choice may therefore benefit from additional qualitative methods, such as interviews and observations. The use of Likert scales introduces potential measurement bias (eg, central tendency, social desirability and acquiescence biases), although inclusion of open-ended questions helped capture additional nuance. This study will inform future work on randomly sampled populations of referees from a wider geographical area. The authors will also explore how patients wish to receive information, including their ability to use different information types to make informed decisions about their referrals.

## Supplementary material

10.1136/bmjhci-2024-101360 online supplemental file 1

10.1136/bmjhci-2024-101360 online supplemental figure 1

10.1136/bmjhci-2024-101360 online supplemental figure 2

## References

[1] General Medical Council (2020). Decision making and consent. https://www.gmc-uk.org/ethical-guidance/ethical-guidance-for-doctors/decision-making-and-consent.

[2] Victoor A, Delnoij DMJ, Friele RD (2012). Determinants of patient choice of healthcare providers: a scoping review. BMC Health Serv Res.

[3] NHS England (2023). Patient choice guidance. https://www.england.nhs.uk/long-read/patient-choice-guidance.

[4] Sabena Isroliwala CW, Sehdev K (2004). A local view of factors influencing patient.

[5] Lewis S, Willis K, Collyer F (2018). Navigating and making choices about healthcare: The role of place. Health Place.

