# Supplementary material for: Exploring factors influencing patient choice in outpatient ophthalmology provider in the North London region: a patient survey
Source: BMJ Health Care Inform. 2025 Oct 29;32(1):e101360. doi: 10.1136/bmjhci-2024-101360 (PMC12574417; doi:10.1136/bmjhci-2024-101360)
Supplement: online supplemental file 1 [file bmjhci-32-1-s001.pdf]

Appendix 1

Free text questions

Q1. When you are referred for care of your eyes in North Central London, what factors would you consider when choosing which care provider you wish to be referred to?

Q2. Reflecting on your past experiences with healthcare in North Central London, how might these inform your future choices?

Q3. When faced with a choice between many care providers in North Central London, how would you go about choosing the one you want to manage your condition?

Q4. What additional local factors do you consider important when choosing a provider in North Central London?

Q5. What material or support could we provide you with that would help you to choose a provider (e.g. patient information leaflets)? What information would you like to see in this material?

Q6. Does the type of care you might need affect your choice of care provider in North Central London? For example, might you choose differently if your referral was likely to lead to an operation compared to a simple clinic appointment?

Q8. Are there any factors not mentioned above that might influence your choice? Please indicate how important any additional factors you mention are to you.

Q9. Finally, having answered all the questions above and thought about what's important to you, please tell us the three factors that are most critical to you when choosing a provider.

Likert scale questions

Q7. Please indicate how important these factors are to you when making a choice between care providers in North Central London. For each statement on the left, you can choose your level of agreement from ""strongly agree"" to ""strongly disagree"". Statements that are most important to you should be marked with ""strongly agree"", and those that are least important to you with ""strongly disagree"".

Q7.1. I prioritize providers based on how soon I can get an appointment.

Q7.2. A nearby location of the healthcare facility is a crucial factor in my decision.

Q7.3. I consider the provider's reputation.

Q7.4. The provider's specialized expertise in my specific health needs is very important to me.

Q7.5. The recommendation of the GP or optometrist who referred me significantly influences my choice.

Q7.6. Recommendations from friends and family significantly influence my choice.

Q7.7. The quality and availability of facilities (e.g., equipment, wards) are important to me.

Q7.8. The professionalism and demeanor of the staff are significant factors in my choice.

Q7.9. The provider's approach to follow-up care and ongoing support is important to me.

Q7.10. Clear and effective communication from the provider is essential.

Q7.11. The environmental footprint and sustainability practices of the provider are important to me.

Q7.12. Good transport links to the healthcare facility are a key consideration for me.

Q7.13. Reviews of the care provider I find online are an important consideration to me.

Q7.14. It is important to me that I have the opportunity to speak to an ophthalmologist (a doctor specialising in the eye) at my appointment.

Q7.15. It is important to me that I have the opportunity to speak to a clinician of any type (e.g. an eye nurse or hospital optometrist) at my appointment.

Q7.16. I am happy to have visits for tests that don't involve speaking to a clinician if I am assured that the quality of care is good.

Q7.17. It is important to me that the provider offers care that is cost-effective for the NHS.

Q7.18. It is important to me that I know how good the clinical outcomes are of patients managed at each provider.
