# Supplementary material for: Exploring factors influencing patient choice in outpatient ophthalmology provider in the North London region: a patient survey
Source: BMJ Health Care Inform. 2025 Oct 29;32(1):e101360. doi: 10.1136/bmjhci-2024-101360 (PMC12574417; doi:10.1136/bmjhci-2024-101360)
Supplement: online supplemental figure 1 [file bmjhci-32-1-s002.pdf]

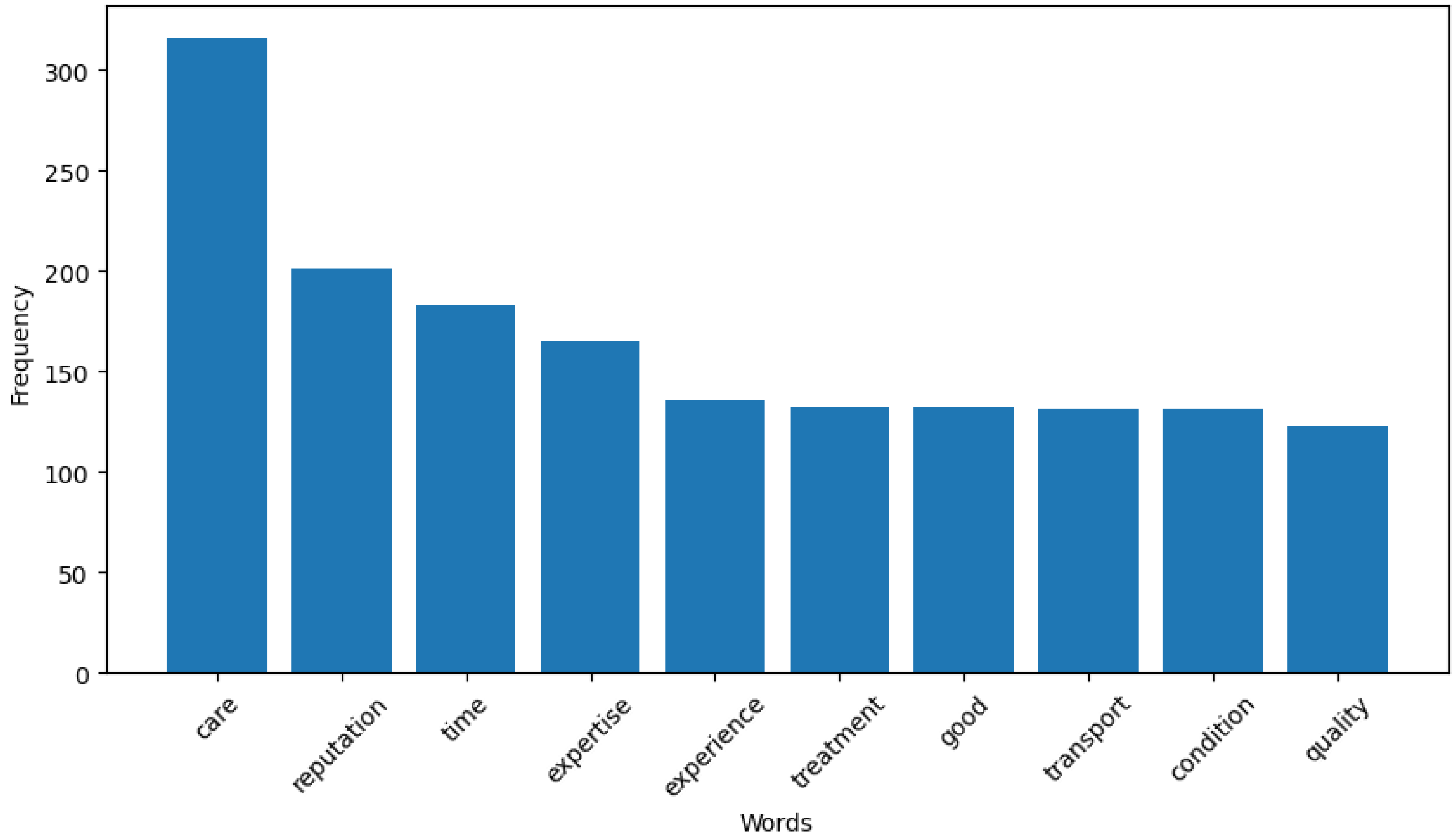

Supplementary Figure 1. Top 10 words by count frequency across free-text response questions, after lemmatisation and stopword removal.
