# Supplementary material for: Exploring factors influencing patient choice in outpatient ophthalmology provider in the North London region: a patient survey
Source: BMJ Health Care Inform. 2025 Oct 29;32(1):e101360. doi: 10.1136/bmjhci-2024-101360 (PMC12574417; doi:10.1136/bmjhci-2024-101360)
Supplement: online supplemental figure 2 [file bmjhci-32-1-s003.pdf]

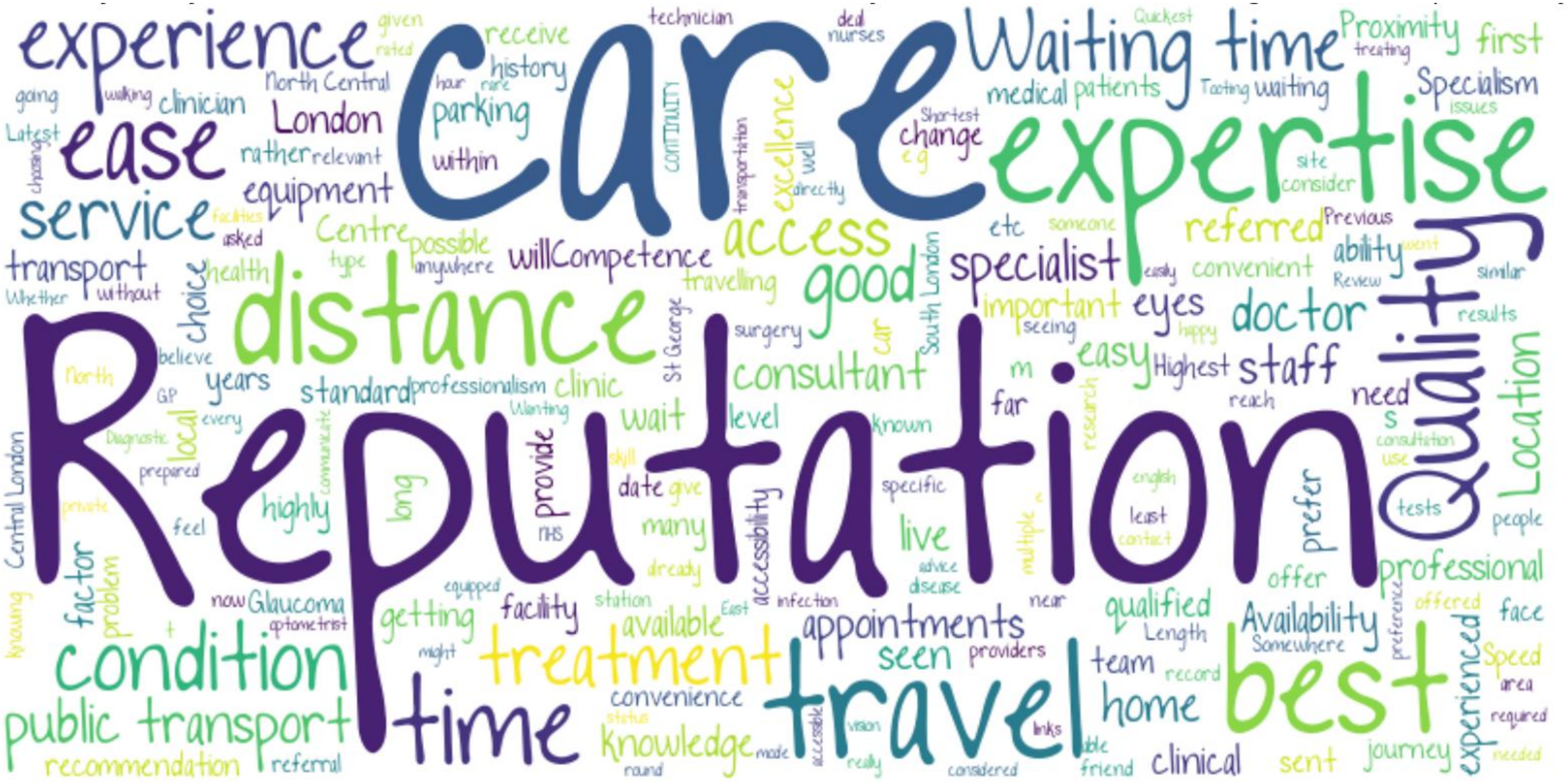

Supplementary figure 2. A word cloud of commonest words to question 1 (“When you are referred for care of your eyes in North Central London, what factors would you consider when choosing which care provider you wish to be referred to?).
